# Supplementary material for: Sex and Cross-Sex Testosterone Treatment Alters Gamma-Hydroxybutyrate Acid Toxicokinetics and Toxicodynamics in Rats
Source: Pharmaceutics. 2024 Jan 21;16(1):143. doi: 10.3390/pharmaceutics16010143 (PMC10821532; doi:10.3390/pharmaceutics16010143)
Supplement: Supplementary file 1 [file pharmaceutics-16-00143-s001.zip › pharmaceutics-2773824-supplementary.pdf]

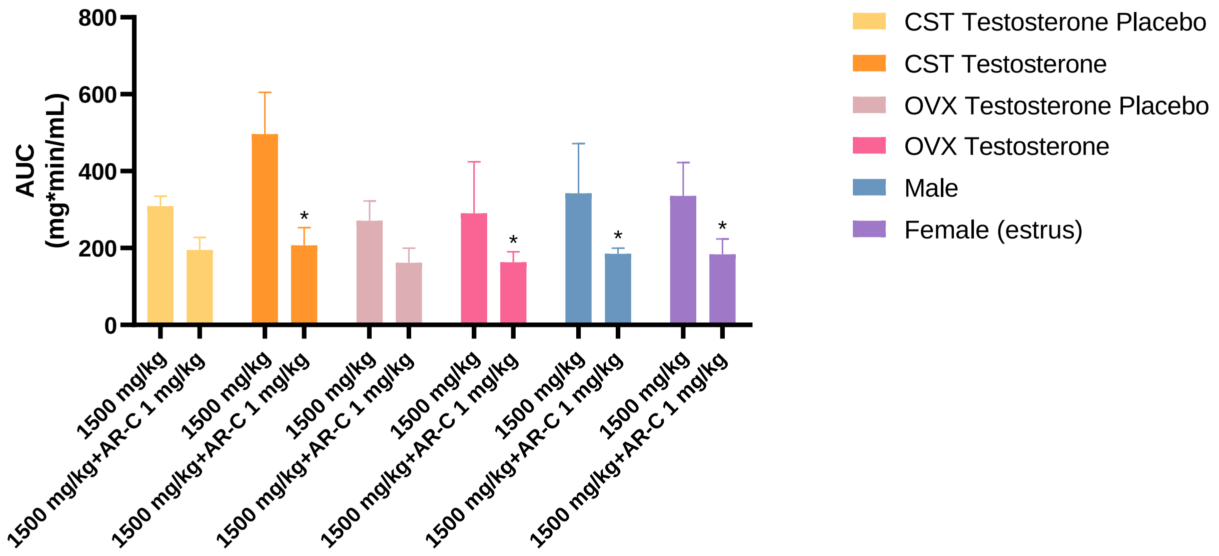

Figure S1. Area under the curve (AUC) following intravenous GHB administration of 1500 mg/kg compared to 1500 mg/kg + 1 mg/kg AR-C 155858. *P < 0.05 compared with 1500 mg/kg of GHB alone. Data are presented as mean ± SD, N = 4-6.

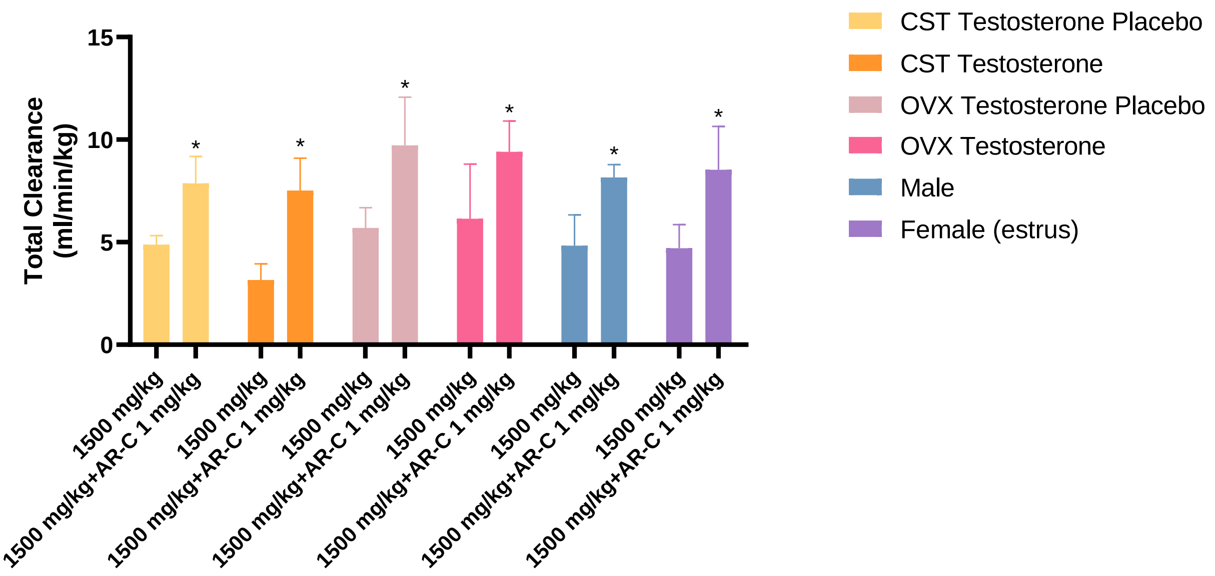

Figure S2. Total clearance following intravenous GHB administration of 1500 mg/kg compared to 1500 mg/kg + 1 mg/kg AR-C 155858. *P < 0.05 compared with 1500 mg/kg of GHB alone. Data are presented as mean ± SD, N = 4-6.

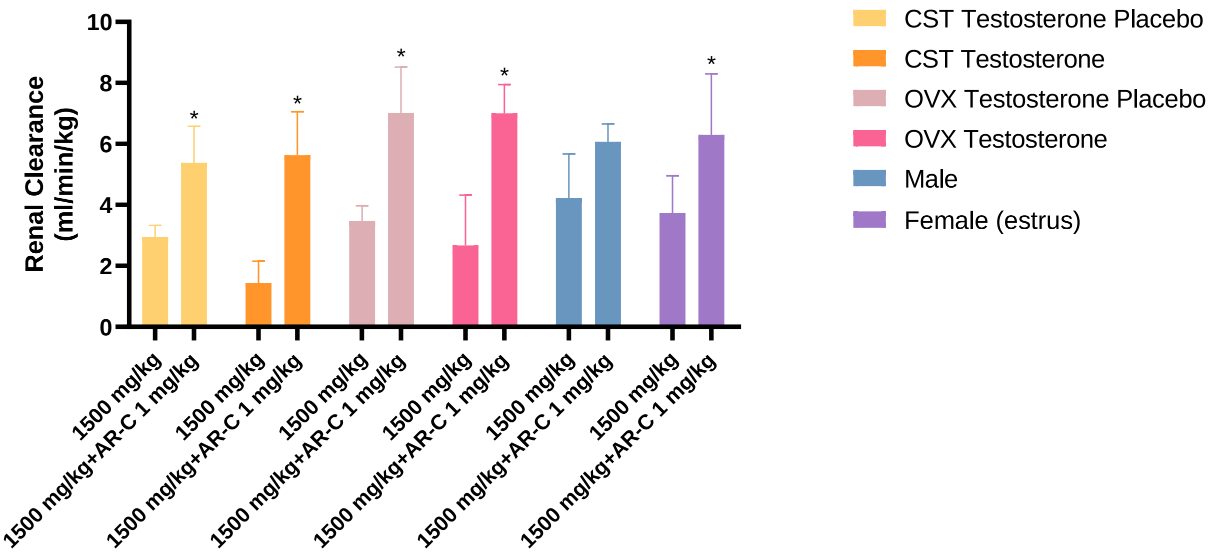

Figure S3. Renal clearance following intravenous GHB administration of 1500 mg/kg compared to 1500 mg/kg + 1 mg/kg AR-C 155858. *P < 0.05 compared with 1500 mg/kg of GHB alone. Data are presented as mean ± SD, N = 4-6.

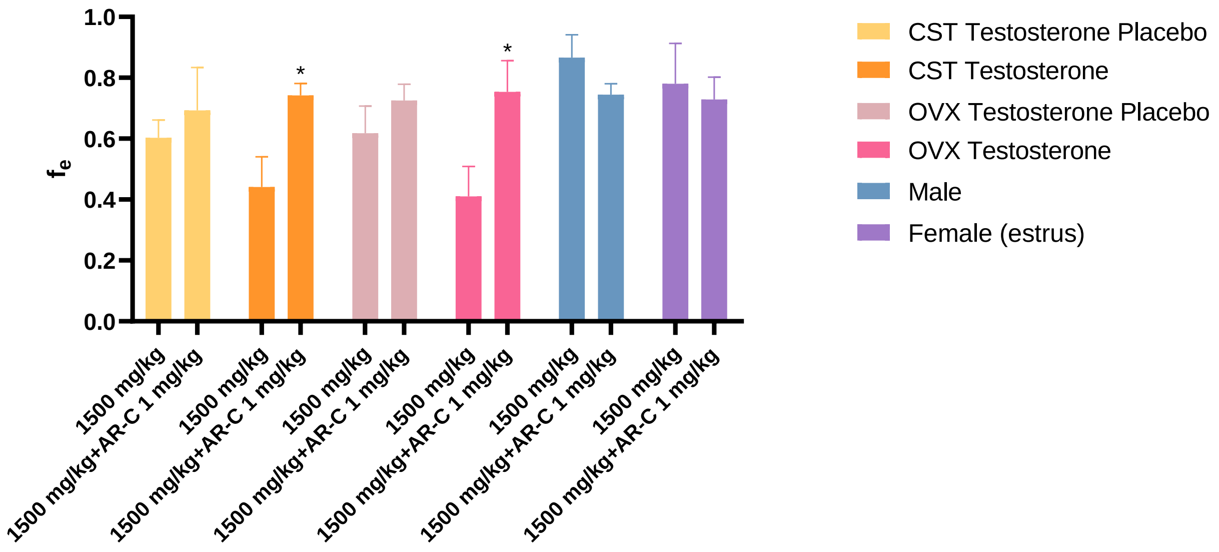

Figure S4. Fraction of drug eliminated in urine (fe) following intravenous GHB administration of 1500 mg/kg compared to 1500 mg/kg + 1 mg/kg AR-C 155858. *P < 0.05 compared with 1500 mg/kg of GHB alone. Data are presented as mean ± SD, N = 4-6.

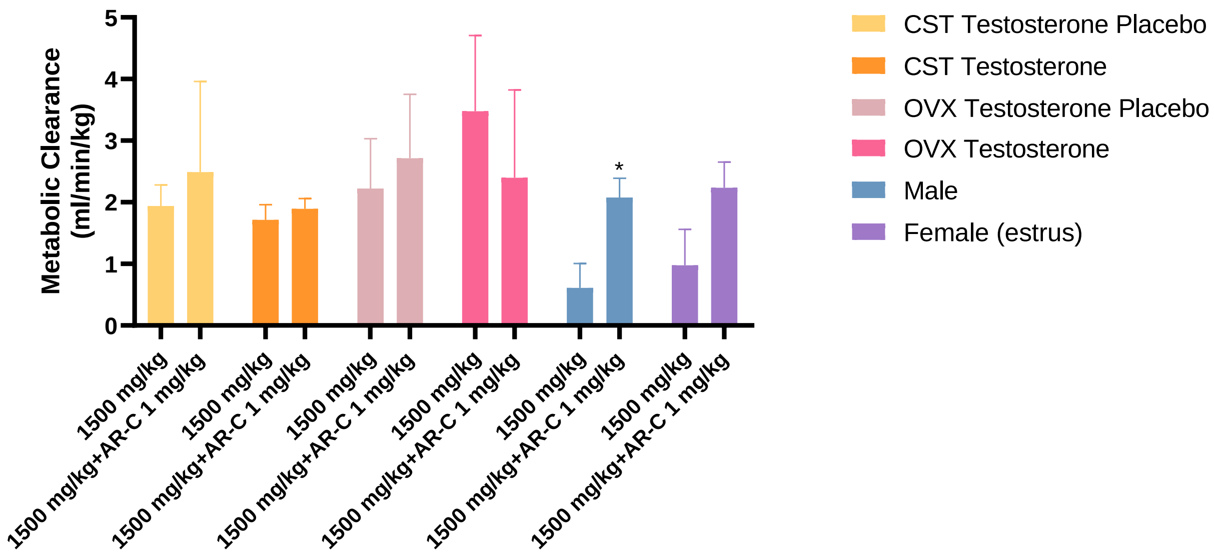

Figure S5. Metabolic clearance following intravenous GHB administration of 1500 mg/kg compared to 1500 mg/kg + 1 mg/kg AR-C 155858. *P < 0.05 compared with 1500 mg/kg of GHB alone. Data are presented as mean ± SD, N = 4-6.

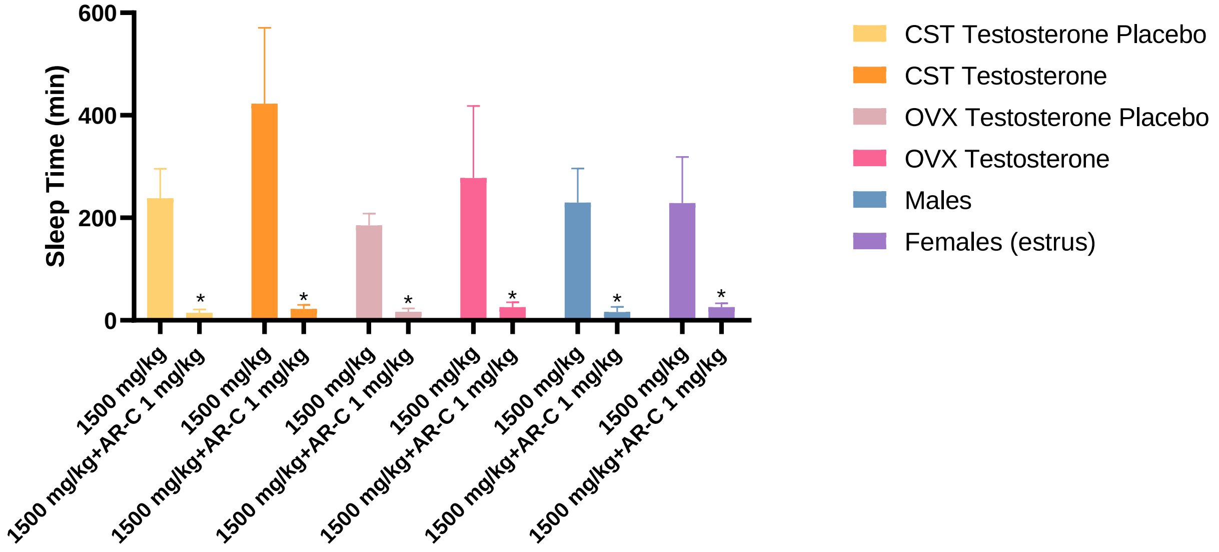

Figure S6. Sedative/hypnotic effect following intravenous GHB administration of 1500 mg/kg compared to 1500 mg/kg + 1 mg/kg AR-C 155858. *P < 0.05 compared with 1500 mg/kg of GHB alone. Data are presented as mean ± SD, N = 5-6.

Table S1 Mass spectrometer parameters of Triple Quad for MRM analysis of GHB.

Parameters	GHB	GHB-d_6
Q1/Q3	105.1/87.1	111.1/93.1
Fragmentors (volts)	55	45
Collision energy	4	4
Cell acceleration voltage (volts)	4	4

Table S2 Mass spectrometer parameters of Triple Quad for MRM analysis of testosterone.

Parameters	Testosterone	Testosterone-d_3
Q1/Q3	289.2/109.1	292.3/109.1
Fragmentors (volts)	112	107
Collision energy	26	26
Cell acceleration voltage (volts)	7	7

Table S3 Toxicokinetic parameters following iv administration of 1000 mg/kg GHB in ovariectomized (OVX) testosterone placebo, OVX testosterone, castrated (CST) testosterone placebo, CST testosterone, intact males and intact females (estrus). Data is presented as mean ± SD, N=5-7.

Parameters	Units	OVX testosterone placebo	OVX testosterone	CST testosterone placebo	CST testosterone	Male	Female (estrus)
AUC	mg*min/mL	181 ± 10.6	169 ± 25.9	222 ± 40.7	197 ± 35.5	224 ± 39.8	150 ± 16.6
CL	mL/min/kg	5.5 ± 0.3	6.0 ± 0.8	4.6 ± 0.8	5.3 ± 1.0	4.6 ± 0.8	6.8 ± 0.7
CL_R	mL/min/kg	2.4 ± 0.1	2.4 ± 0.7	3.0 ± 0.7	3.2 ± 0.9	2.2 ± 0.4	2.6 ± 0.5
fe		0.29 ± 0.01	0.26 ± 0.05	0.43 ± 0.05	0.40 ± 0.07	0.48 ± 0.06	0.39 ± 0.07
CL_m	mL/min/kg	3.1 ± 0.3	3.6 ± 0.6	1.6 ± 0.5	2.1 ± 0.4	2.4 ± 0.5	4.1 ± 0.6

AUC - Area under the curve of plasma concentration versus time profile

CL - Total clearance

CL_R - Renal clearance

fe – The fraction of drug eliminated from urine

CLm – Metabolic clearance

Table S4 Toxicokinetic parameters following iv administration of 1500 mg/kg GHB in ovariectomized (OVX) testosterone placebo, OVX testosterone, castrated (CST) testosterone placebo, CST testosterone, intact males and intact females (estrus). Data is presented as mean ± SD, N=5-6.

Parameters	Units	OVX testosterone placebo	OVX testosterone	CST testosterone placebo	CST testosterone	Male	Female (estrus)
AUC	mg*min/mL	271 ± 47.0	290 ± 122.7	309 ± 23.8	496 ± 97.6	343 ± 117.8	336 ± 78.8
CL	mL/min/kg	5.7 ± 0.9	6.1 ± 2.4	4.9 ± 0.4	3.2 ± 0.7	4.8 ± 1.4	4.7 ± 1.1
CL_R	mL/min/kg	3.5 ± 0.5	2.7 ± 1.5	2.9 ± 0.4	1.4 ± 0.6	4.2 ± 1.3	3.7 ± 1.1
fe		0.62 ± 0.08	0.41 ± 0.09	0.60 ± 0.05	0.44 ± 0.09	0.87 ± 0.07	0.78 ± 0.12
CL_m	mL/min/kg	2.2 ± 0.7	3.5 ± 1.1	1.9 ± 0.3	1.7 ± 0.2	0.6 ± 0.4	1.0 ± 0.5

Table S5 Toxicokinetic parameters following iv administration of 1500 mg/kg GHB + 1mg/kg AR-C 155858 in ovariectomized (OVX) testosterone placebo, OVX testosterone, castrated (CST) testosterone placebo, CST testosterone, intact males and intact females (estrus). Data is presented as mean ± SD, N=4-6.

Parameters	Units	OVX testosterone placebo	OVX testosterone	CST testosterone placebo	CST testosterone	Male	Female (estrus)
AUC	mg*min/mL	162 ± 34.8	163 ± 24.9	195 ± 29.6	207 ± 40.4	185 ± 13.2	184 ± 36.3
CL	mL/min/kg	9.7 ± 2.1	9.4 ± 1.4	7.9 ± 1.2	7.5 ± 1.4	8.2 ± 0.6	8.5 ± 1.9
CL_R	mL/min/kg	7.0 ± 1.4	7.0 ± 0.9	5.4 ± 1.1	5.6 ± 1.2	6.1 ± 0.5	6.3 ± 1.8
fe		0.73 ± 0.05	0.75 ± 0.09	0.69 ± 0.13	0.74 ± 0.03	0.74 ± 0.03	0.73 ± 0.07
CL_m	mL/min/kg	2.7 ± 0.9	2.4 ± 1.3	2.5 ± 1.3	1.9 ± 0.1	2.1 ± 0.3	2.2 ± 0.4
